# Why the Rationale for Canine *Borrelia burgdorferi* Vaccination Is Unpersuasive

**DOI:** 10.3389/fvets.2021.719060

**Published:** 2021-08-11

**Authors:** Nadine A. Vogt, Christian P. G. Stevens

**Affiliations:** ^1^Department of Population Medicine, Ontario Veterinary College, University of Guelph, Guelph, ON, Canada; ^2^Department of Philosophy, King's College London, London, United Kingdom

**Keywords:** Lyme, *Borrelia*, vaccine, canine, dog

## Introduction

Canine *Borrelia burgdorferi* vaccines have long been a bone of contention in the veterinary profession (1, 2). Proponents of this non-core vaccine maintain that it is important to provide protective immunity to dogs exposed to tick-infested areas. While *B. burgdorferi* vaccines were never intended to replace tick preventives, in cases of poor compliance with product administration or product failure, pet owners can rest assured that their animal is protected against Lyme borreliosis. In the 2018 small animal consensus statement by the American College of Veterinary Internal Medicine, however, experts did not reach a consensus on whether or not to recommend the vaccine: three were in favor, and three were against (3). According to the vaccination guidelines from the American Animal Hospital Association (AAHA), *not generally recommended* vaccines are ones for “diseases of low clinical significance or that respond readily to treatment; vaccines for which evidence of efficacy in the field is minimal; or vaccines that may produce a relatively higher incidence of adverse events with limited benefit” (4). In this brief critical discussion, we argue that *B. burgdorferi* vaccines in dogs meet all of the criteria outlined by AAHA for a vaccine that is *not generally recommended*, and that these vaccines do not confer any clear benefit to public health, as Lyme borreliosis is a vector-borne disease. For these reasons, we conclude that the rationale for canine *B*. *burgdorferi* vaccination is unpersuasive.

## Disease of Low Clinical Significance

The vast majority of dogs appear to exhibit a kind of natural immunity to Lyme borreliosis; roughly 95% of dogs remain asymptomatic following exposure to a tick infected with *B. burgdorferi* (5). Although there are reports of potentially fatal cardiac and renal sequelae associated with *B. burgdorferi* in the literature (6–10), these syndromes appear to be exceedingly rare. Furthermore, these purported syndromes are challenging to conclusively link with Lyme borreliosis for that same reason (rarity of disease), as well as a lack of disease model, with no gold standard test to differentiate between clinical and incidental infections (11). It seems fair to say, then, that Lyme borreliosis meets the first criterion of AAHA of being a “disease of low clinical significance.”

## Effective Tick Preventives and Good Response to Antimicrobial Treatment

As many researchers and veterinarians in clinical practice have noted over the years, prompt treatment of clinically affected dogs with antibiotics is reported to be highly successful (3, 12). It would appear, then, that Lyme borreliosis is not only a “disease of low clinical significance,” but it also “responds readily to treatment,” as per the latter part of the first criterion of AAHA. Leaving dogs susceptible to tick exposures, however, is undesirable, since ticks can harbor a number of pathogenic infectious agents other than *B. burgdorferi*, such as *Anaplasma, Ehrlichia*, and *Rickettsia* spp. Tick preventives, which are shown to be highly effective in preventing the attachment and subsequent feeding of ticks (13–15), are arguably the gold standard of prevention against *Borrelia* infection and other tick-borne diseases.

If a tick prevention regimen is properly adhered to, there should be no need for vaccination. It is occasionally argued that *B. burgdorferi* vaccines are still to be recommended in cases where owner compliance with tick preventive regimens may be an issue. A plausible rationale for recommending a vaccine, however, requires good evidence of safety and efficacy. Concerns about the safety and efficacy of *B. burgdorferi* vaccines have previously been raised, and these topics remain controversial (2, 3, 10, 16).

## Questionable Vaccine Efficacy

Several different types of *B. burgdorferi* vaccines are currently commercially available, including several bacterins (e.g., LymeVax^®^, Zoetis; Nobivac^®^ Lyme, Merck Animal Health), recombinant OspA subunit vaccines (e.g., RECOMBITEK^®^ Lyme, Boehringer Ingelheim), and a chimeric recombinant OspA and OspC vaccine (VANGUARD^®^ crLyme, Zoetis). To date, there are no available experimental field trials examining the efficacy of canine *B. burgdorferi* vaccines (17). A previous systematic review examining the efficacy of canine *B. burgdorferi* vaccines determined that the existing literature consisted of observational studies and challenge trials [i.e., experimental studies where disease is deliberately induced; (17)]. Notably, all three observational studies included in the systematic review suffer from a serious study design defect: none accounted for the use of tick preventives, which, by itself, could be responsible for the differences in infection rates between vaccinates and control group dogs (18–20). Thus, the second criterion of AAHA is also plausibly fulfilled: “evidence of [vaccine] efficacy in the field is minimal.” It is also worth noting that *B. burgdorferi* vaccines are not labeled to provide protection against other species of *Borrelia* which are known to infect and cause clinical illness in dogs in Europe [e.g., *Borrelia afzelii, Borrelia garinii*; (21)].

## Vaccine Safety Concerns

A retrospective observational study of over 1 million dogs demonstrated that the incidence of acute adverse events in dogs within 3 days was highest for those receiving a *B. burgdorferi* bacterin vaccine (i.e., LymeVax^®^ 43.7/10,000 doses) compared with all other vaccines examined (22). Studies evaluating the field safety of canine *B. burgdorferi* vaccines documenting minimal adverse events have limited generalizability, as only acute adverse events up to 10 days post-vaccination were evaluated (19, 22, 23). The risk of more latent adverse events has not been specifically examined for canine *B. burgdorferi* vaccines. A final consideration is that a relatively short duration of immunity necessitating annual revaccination exposes dogs to potential vaccine-related adverse events every year (24, 25). In conclusion, it is plausible that the third criterion of AAHA is also satisfied: *B. burgdorferi* “vaccines [.] may produce a relatively higher incidence of adverse events with limited benefit.”

## Discussion: A One Health Perspective

Aside from the protection of the health of an individual animal, there are other good reasons for recommending a vaccine, including for the protection of public health, and/or to generate herd immunity, with the intent to limit or entirely curb the spread of disease. Canine kennel cough vaccines, for example, do not only protect the health of an individual dog but also prevent the transmission of bacteria (e.g., *Bordetella*) and viruses (e.g., adenovirus, parainfluenza) to other susceptible dogs within a high-density environment. This stands in contrast to *B. burgdorferi*, where infected dogs cannot directly transmit the infectious agent to other dogs: Lyme borreliosis is a vector-borne disease that requires a tick to carry and transmit the infectious agent. Thus, vaccination of an individual dog with a *B. burgdorferi* vaccine neither protects against direct transmission to other dogs, nor to humans. It is occasionally suggested that *B. burgdorferi* vaccines have utility in neutralizing infected ticks (26), which could, theoretically, help curb the spread of Lyme borreliosis in both humans and animals alike. In our view, the “vaccination” of ticks through their feeding on vaccinated dogs is unlikely to put any serious dent into the prevalence of *B. burgdorferi* in tick populations, since this spirochetal bacterium is geographically widespread, endemic to many regions and self-sustaining in a sylvatic transmission cycle, independent of dogs (27).

In terms of a risk-benefit analysis for the individual animal, we argue that the risks associated with *B. burgdorferi* vaccines are not outweighed by their benefit, as the vast majority of dogs demonstrate a natural immunity to *B. burgdorferi*, in contrast to other pathogenic agents. Moreover, in the subpopulation of dogs prone to developing Lyme borreliosis following exposure to *B. burgdorferi*, we cannot, at this time, rule out the possibility that this is a subpopulation of dogs with dysfunctional immune systems who may be at increased risk of having an adverse reaction to a *B. burgdorferi* vaccine (3). Future research characterizing the demographic and immunological characteristics of these canine subpopulations may shed light on this issue.

In line with AAHA guidelines, we have argued that vaccination of dogs for Lyme borreliosis is unwarranted, since clinical disease is rare, cases respond readily to treatment, alternative prevention methods are effective, and vaccine efficacy and safety are questionable. If this is correct, then it is in the best interests of our canine companions, and not contrary to human or canine herd health, to avoid the administration of canine *B. burgdorferi* vaccines at this time.

## Author Contributions

NV and CS co-wrote the manuscript and contributed equally to the intellectual content. Both authors contributed to the article and approved the submitted version.

## Conflict of Interest

The authors declare that the research was conducted in the absence of any commercial or financial relationships that could be construed as a potential conflict of interest.

## Publisher's Note

All claims expressed in this article are solely those of the authors and do not necessarily represent those of their affiliated organizations, or those of the publisher, the editors and the reviewers. Any product that may be evaluated in this article, or claim that may be made by its manufacturer, is not guaranteed or endorsed by the publisher.
